# Supplementary material for: Epipyrone A, a Broad-Spectrum Antifungal Compound Produced by Epicoccum nigrum ICMP 19927
Source: Molecules. 2020 Dec 18;25(24):5997. doi: 10.3390/molecules25245997 (PMC7766273; doi:10.3390/molecules25245997)
Supplement: Supplementary file 1 [file molecules-25-05997-s001.zip › molecules-1003488-supplementary.docx]

Supporting Information

Epipyrone A, a broad-spectrum antifungal compound produced by *Epicoccum nigrum* ICMP 19927

Alex J. Lee^1^, Melissa M. Cadelis^2^, Sang H. Kim^1^, Simon Swift^3^, Brent R. Copp^2^, and Silas G. Villas-Boas^1^*

^1^ School of Biological Sciences, University of Auckland, 3A Symonds Street, 1010 Auckland, New Zealand

^2^ School of Chemical Sciences, University of Auckland, 23 Symonds Street, 1010 Auckland, New Zealand

^3^ School of Medical Sciences, University of Auckland, 85 Park Road, Grafton, 1023 Auckland, New Zealand

***** Correspondence: s.villas-boas@auckland.ac.nz

**Contents**

| **Figure S1**. ^1^H NMR spectrum (CD_3_OD, 500 MHz) of **1** | **S2** |
| --- | --- |
| **Figure S2**. ^13^C NMR spectrum (CD_3_OD, 125 MHz) of **1** | **S3** |
| **Figure S3**. COSY NMR spectrum (CD_3_OD) of **1** | **S4** |
| **Figure S4**. HSQC NMR spectrum (CD_3_OD) of **1** | **S5** |
| **Figure S5**. HMBC NMR spectrum (CD_3_OD) of **1** | **S6** |
| **Figure S6**. ROESY NMR spectrum (CD_3_OD) of **1** | **S7** |
| **Figure S7**. ^13^C NMR spectrum (CD_3_OD, 125 MHz) of [U-^13^C]glucose labelled **1** | **S8** |
| **Figure S8**. ^13^C-^13^C COSY NMR spectrum (CD_3_OD) of [U-^13^C]glucose labelled **1** | **S9** |
| **Figure S9**. HRESIMS of **1** | **S10** |
| **Figure S10**. ^1^H NMR spectrum (CD_3_OD, 500 MHz) of **2** | **S11** |
| **Figure S11**. ^13^C NMR spectrum (CD_3_OD, 125 MHz) of [U-^13^C]glucose labelled **2** | **S12** |
| **Figure S12**. ^13^C-^13^C COSY NMR spectrum (CD_3_OD) of [U-^13^C]glucose labelled **2** | **S13** |


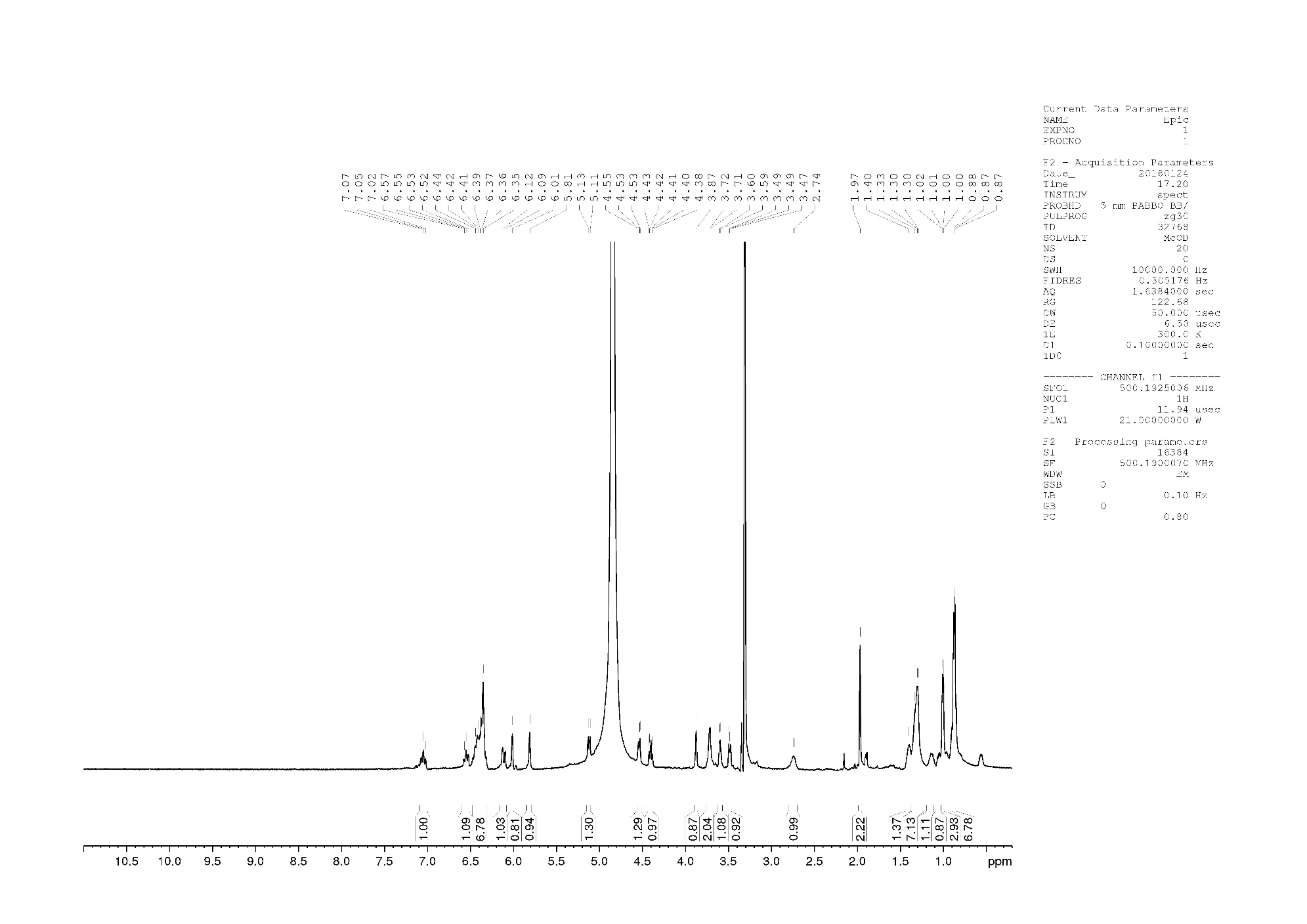


**Figure S1**. ^1^H NMR spectrum (CD_3_OD, 500 MHz) of **1**


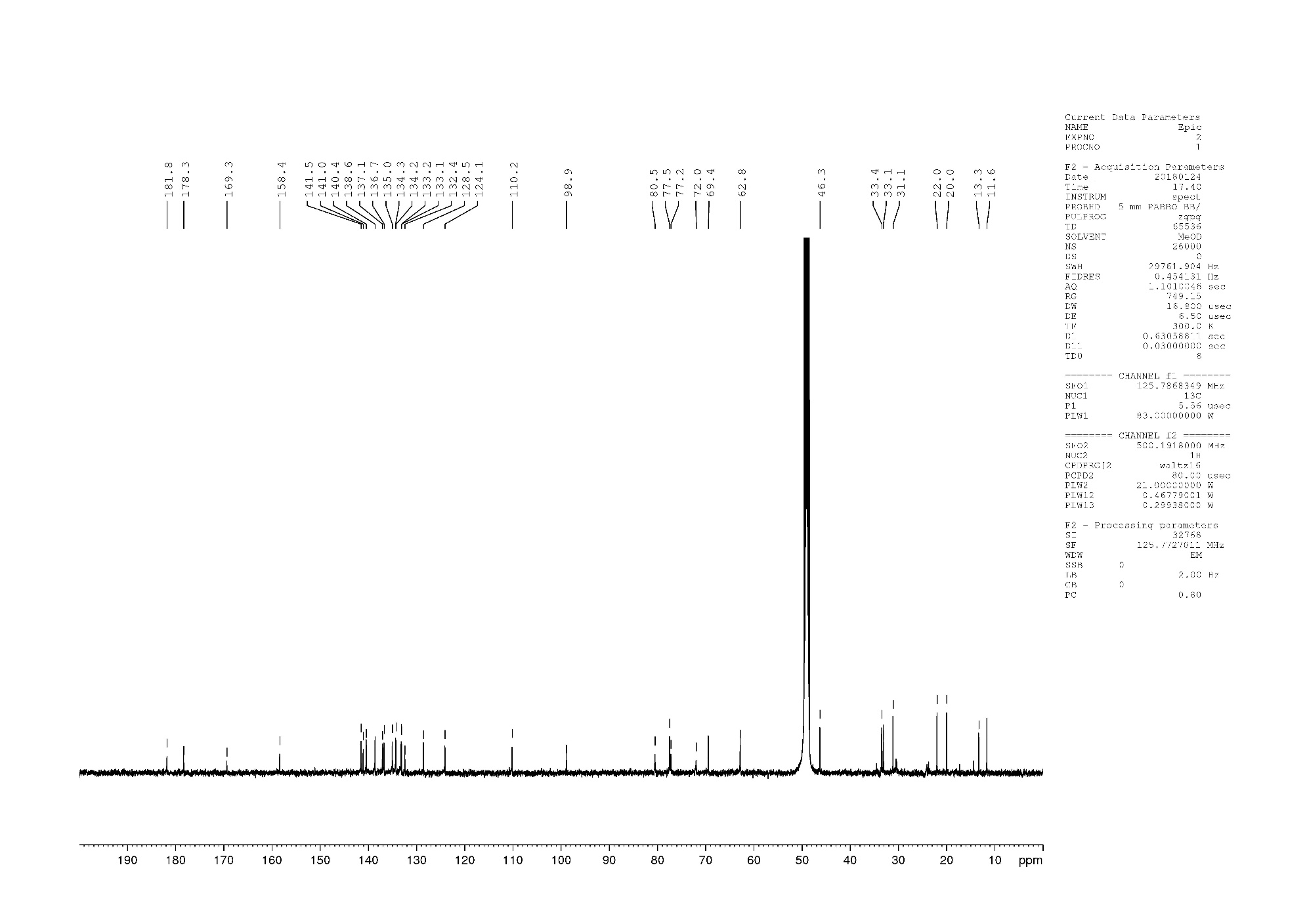


**Figure S2**. ^13^C NMR spectrum (CD_3_OD, 125 MHz) of **1**


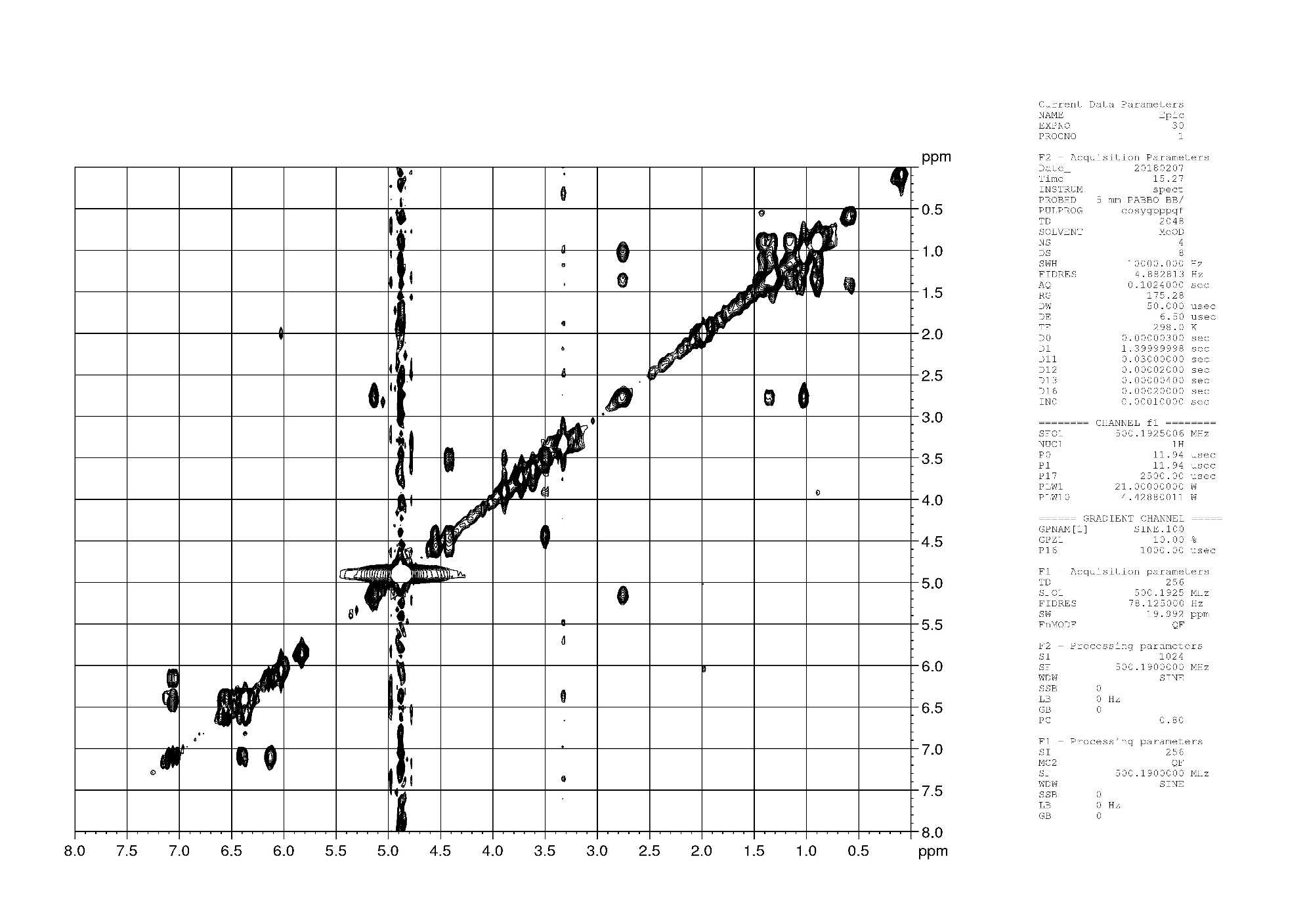


**Figure S3**. COSY NMR spectrum (CD_3_OD) of **1**


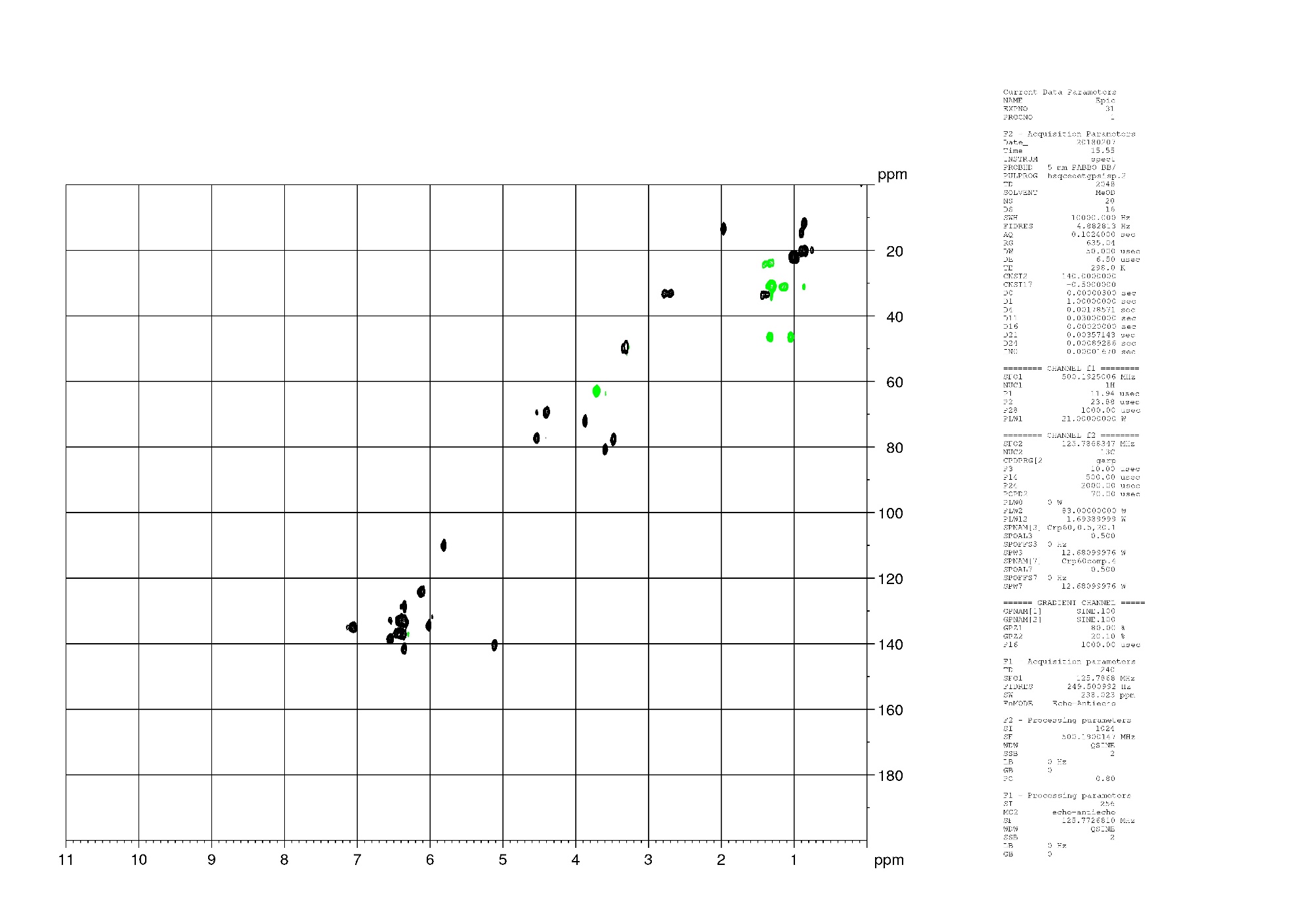


**Figure S4**. HSQC NMR spectrum (CD_3_OD) of **1**


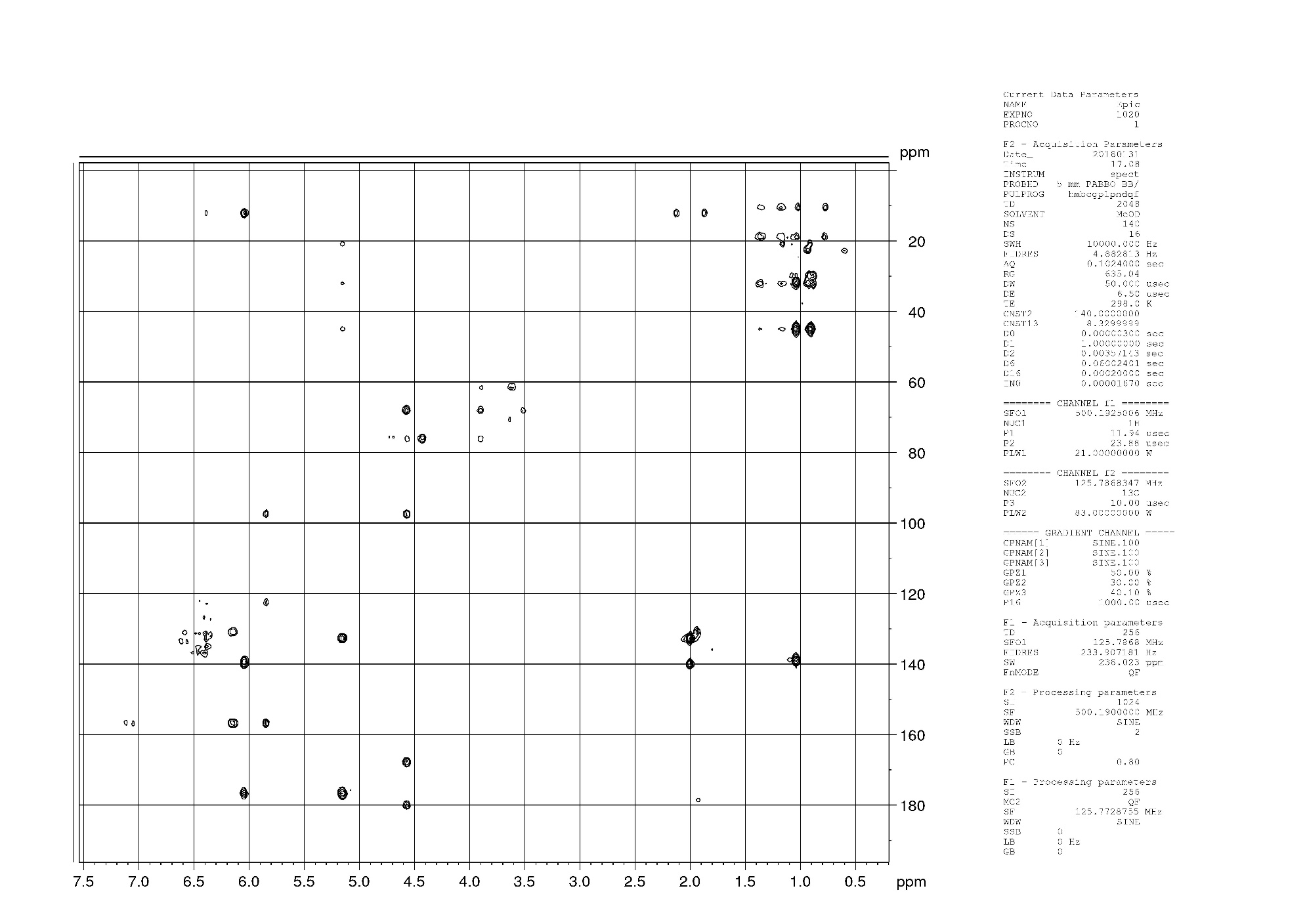


**Figure S5**. HMBC NMR spectrum (CD_3_OD) of **1**


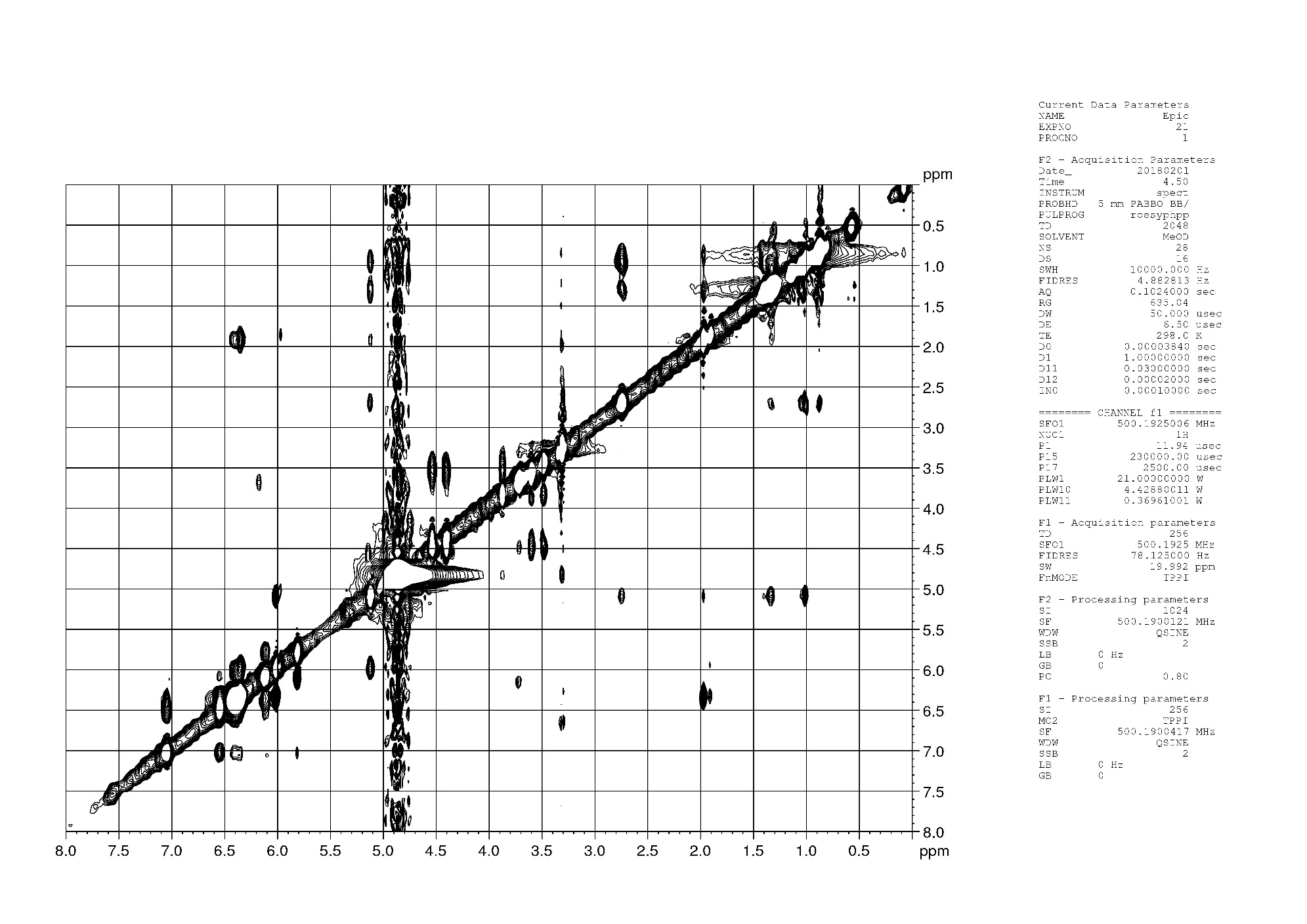


**Figure S6**. ROESY NMR spectrum (CD_3_OD) of **1**


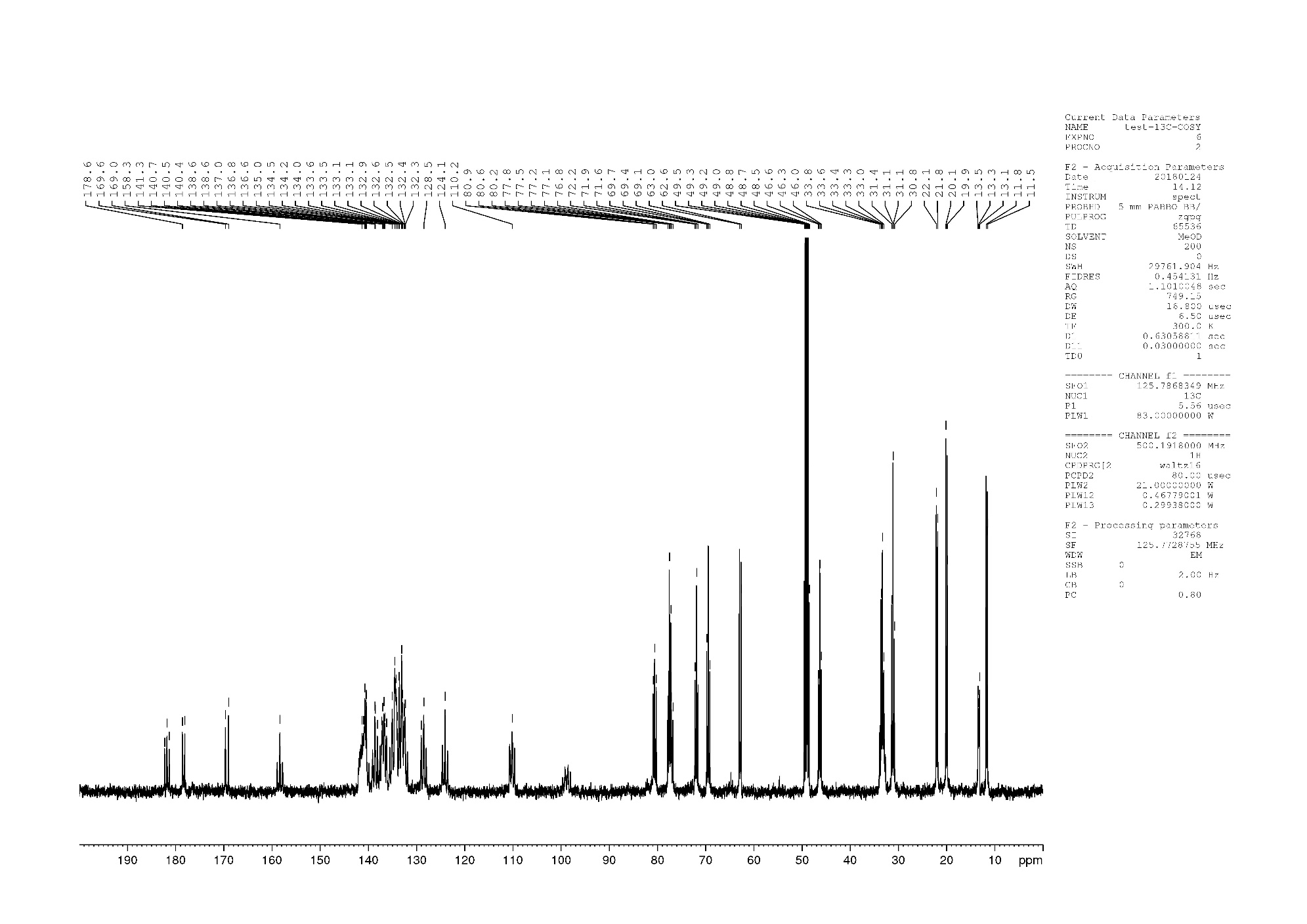


**Figure S7**. ^13^C NMR spectrum (CD_3_OD, 125 MHz) of [U-^13^C]glucose labelled **1**


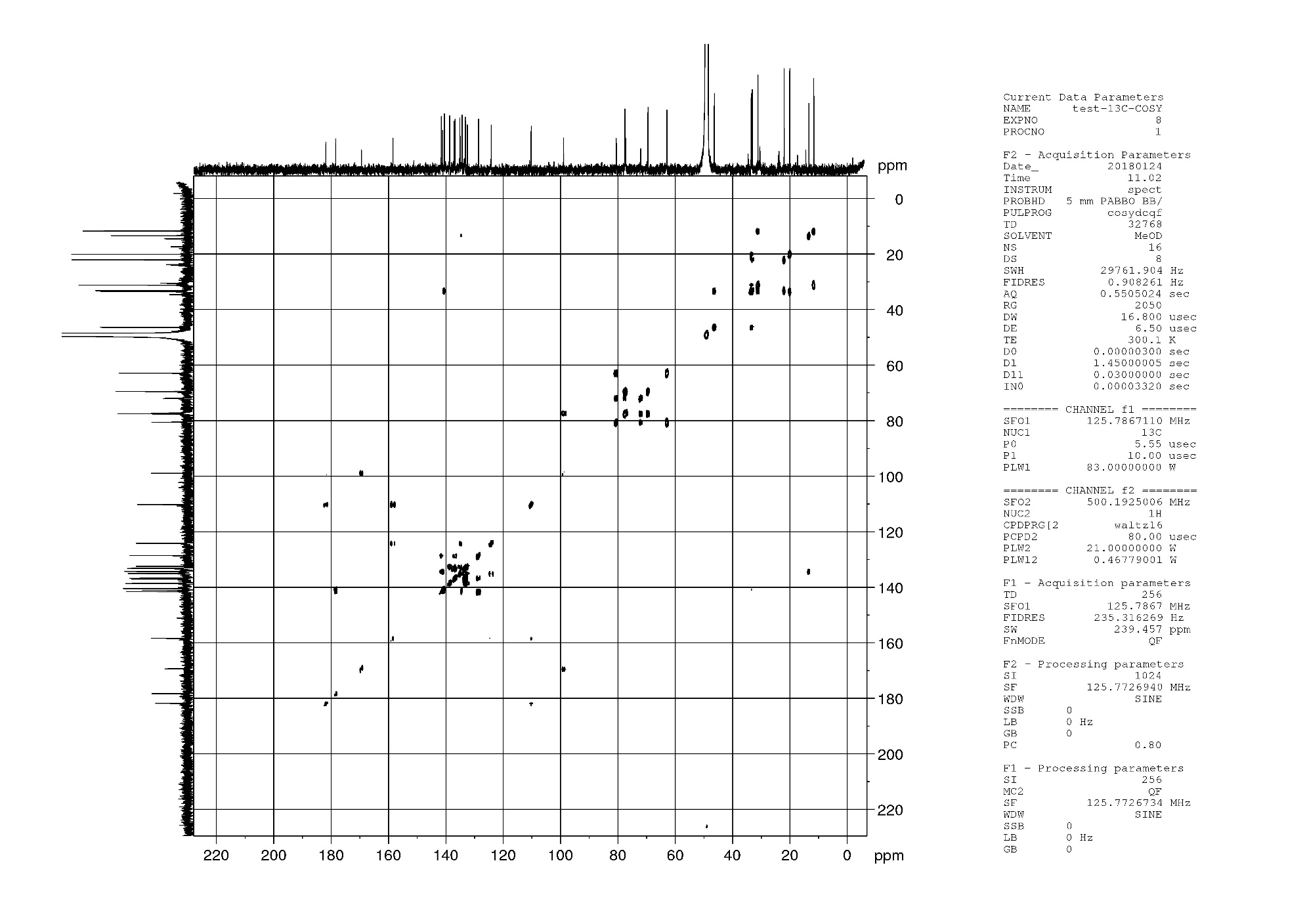


**Figure S8**. ^13^C-^13^C COSY NMR spectrum (CD_3_OD) of [U-^13^C]glucose labelled 1


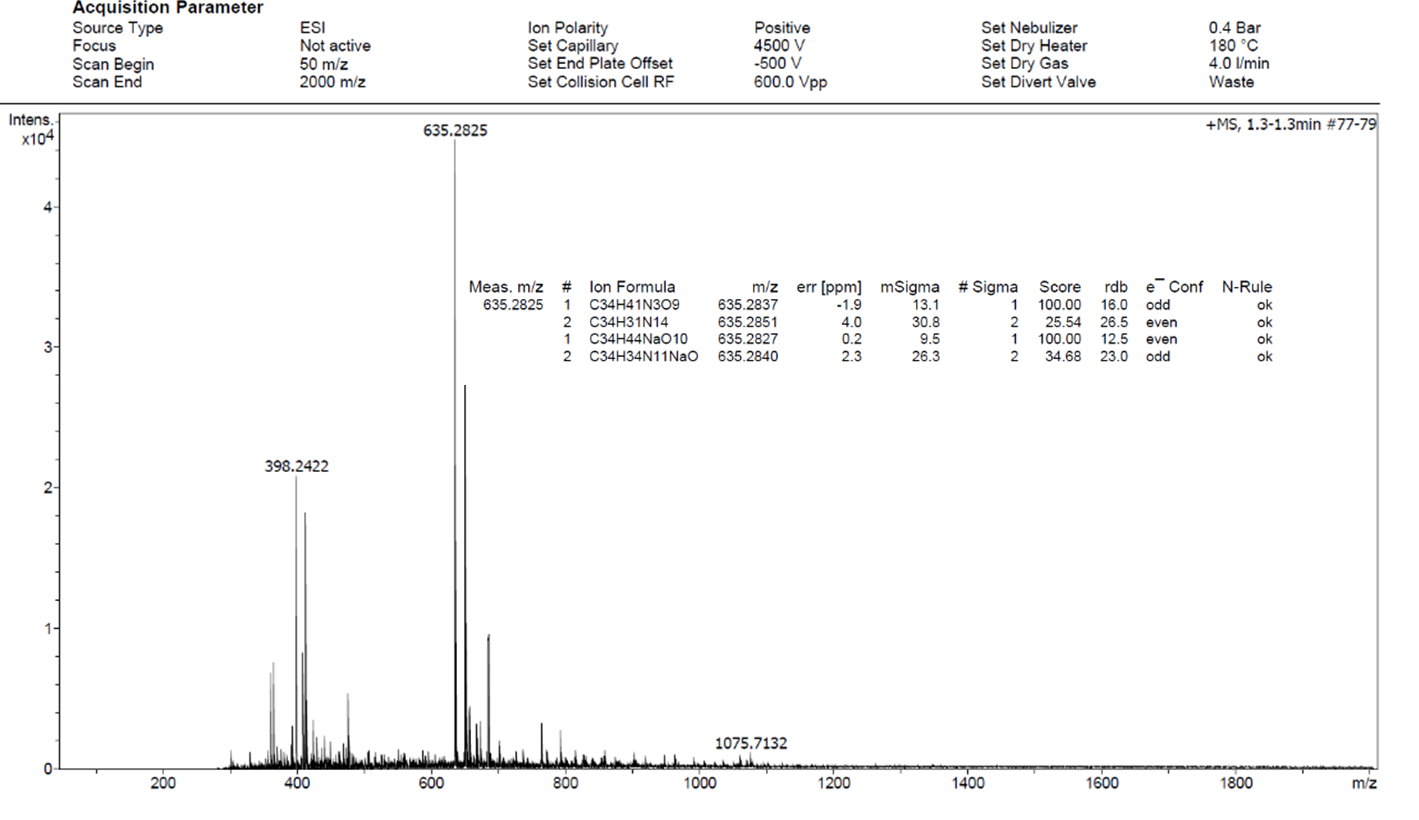


**Figure S9**. HRESIMS of **1**


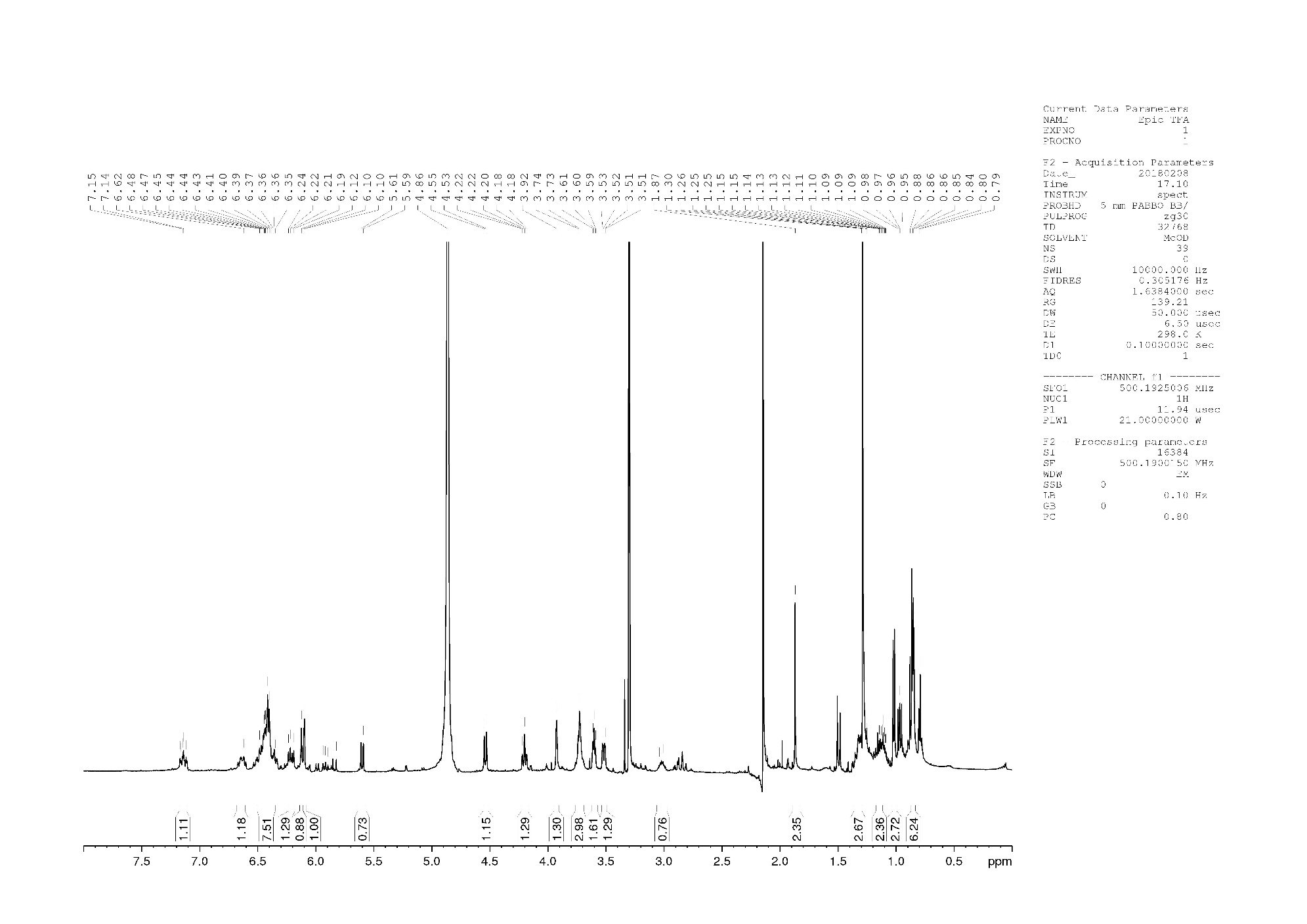


**Figure S10**. ^1^H NMR spectrum (CD_3_OD, 500 MHz) of **2**


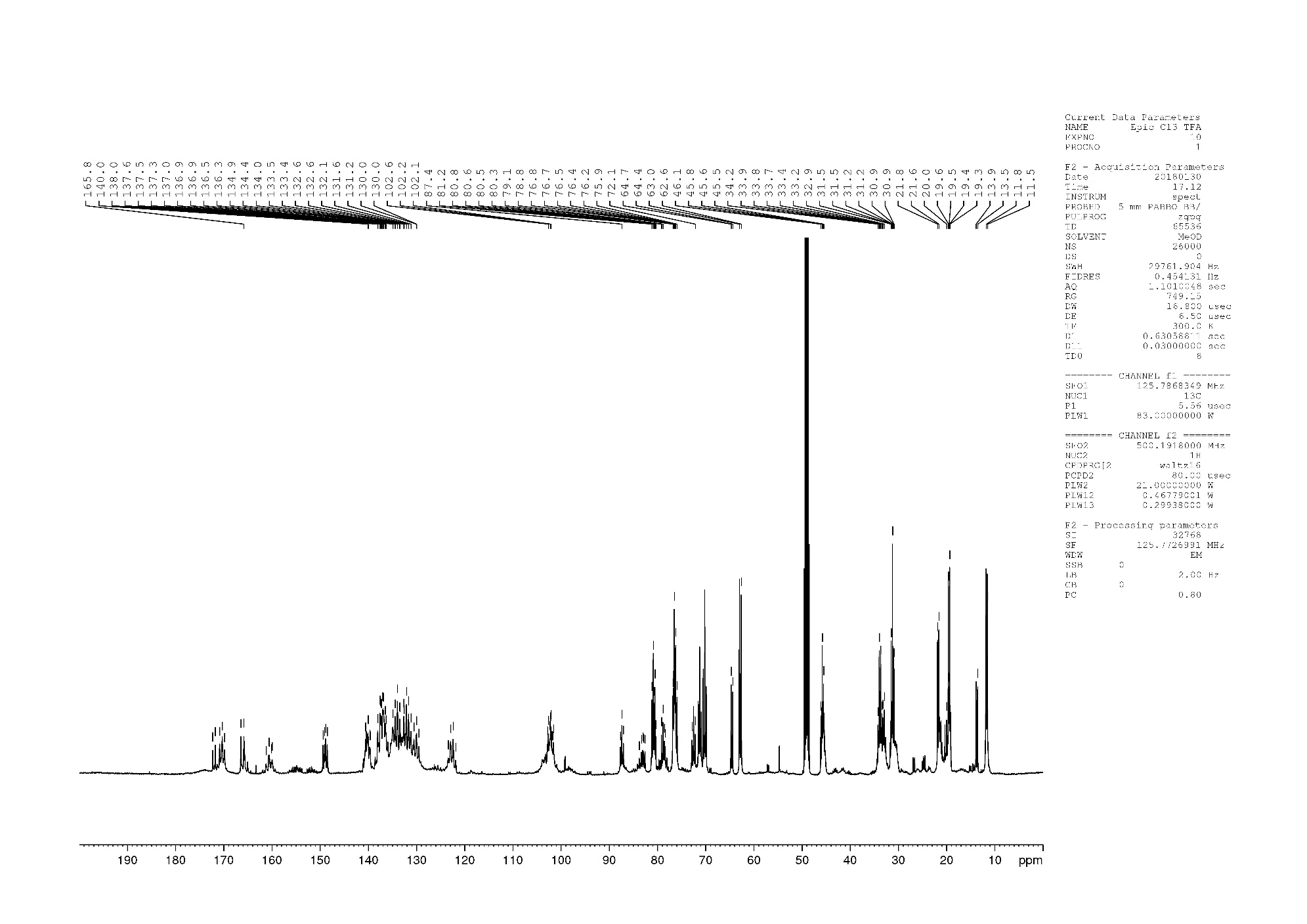


**Figure S11**. ^13^C NMR spectrum (CD_3_OD, 125 MHz) of [U-^13^C]glucose labelled **2**


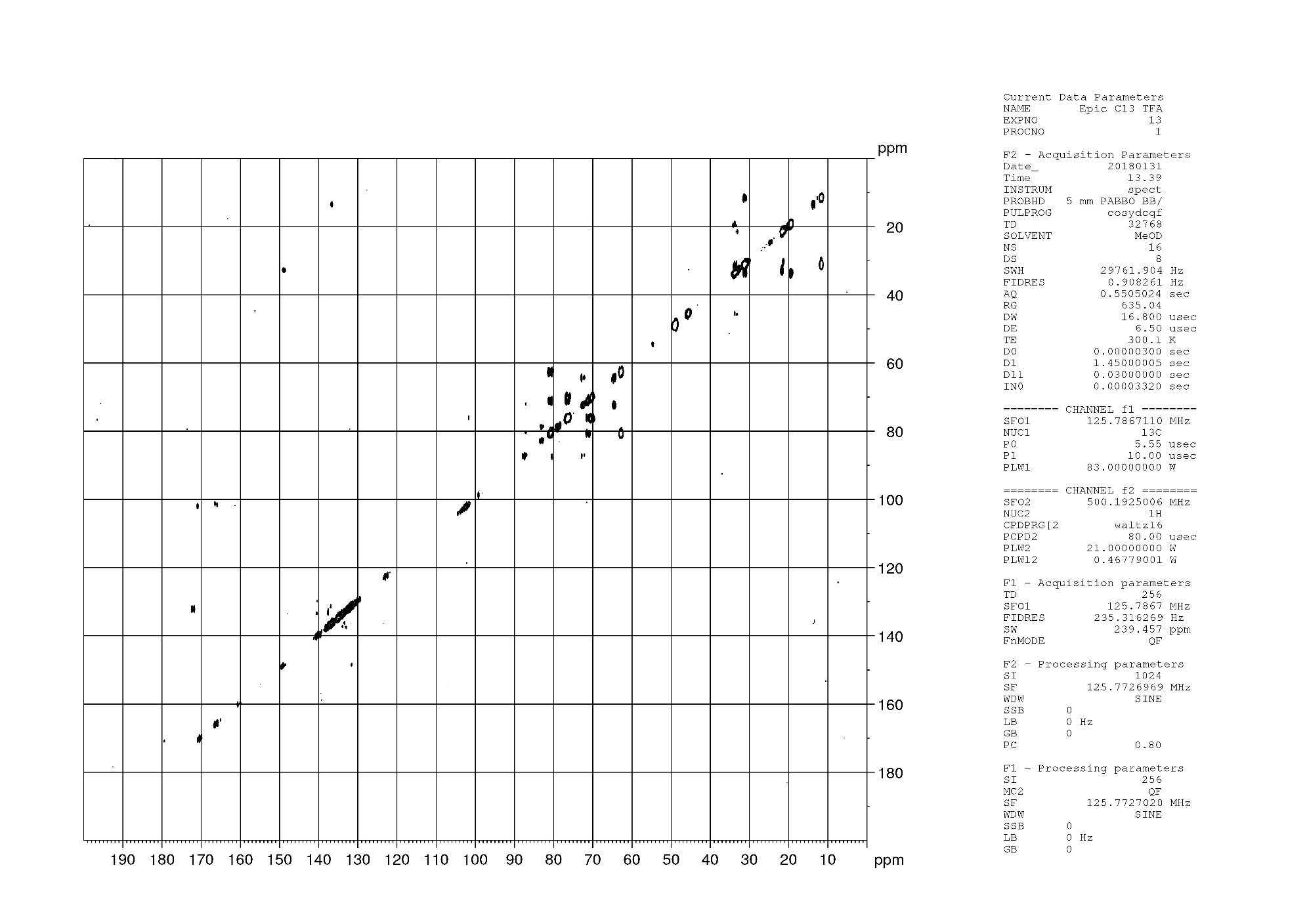


**Figure S12**. ^13^C-^13^C COSY NMR spectrum (CD_3_OD) of [U-^13^C]glucose labelled **2**
